# A New Method for Inversion of Dam Foundation Hydraulic Conductivity Using an Improved Genetic Algorithm Coupled with an Unsaturated Equivalent Continuum Model and Its Application

**DOI:** 10.3390/ma16041662

**Published:** 2023-02-16

**Authors:** Jiayi Peng, Zhenzhong Shen, Liqun Xu, Lei Gan, Jiacheng Tan

**Affiliations:** 1 College of Water Conservancy and Hydropower Engineering, Hohai University, Nanjing 210098, China; 2 State Key Laboratory of Hydrology–Water Resources and Hydraulic Engineering, Hohai University, Nanjing 210098, China

**Keywords:** inversion of hydraulic conductivity, improved genetic algorithm, unsaturated equivalent continuum model, concrete-face rockfill dam, cement grouting curtain

## Abstract

Seepage is a main cause of dam failure, and its stability analysis is the focus of a dam’s design, construction, and management. Because a geological survey can only determine the range of a dam foundation’s hydraulic conductivity, hydraulic conductivity inversion is crucial in engineering. However, current inversion methods of dam hydraulic conductivity are either not accurate enough or too complex to be directly used in engineering. Therefore, this paper proposes a new method for the inversion of hydraulic conductivity with high application value in hydraulic engineering using an improved genetic algorithm coupled with an unsaturated equivalent continuum model (IGA–UECM). This method is implemented by a new code that fully considers engineering applicability. In addition to overcoming the premature convergence shortcomings of traditional genetic algorithms, it converges faster than Bayesian optimization and tree-structured Parzen estimator inversion algorithms. This method is verified by comparing the water head from drilling exploration and inversion. The results of the inversion are used to study the influence of a cement grouting curtain layout scheme on the seepage field of the Hami concrete-face rockfill dam in China, which is used as an engineering application case of the IGA–UECM. The law of the seepage field is reasonable, which verifies the validity of the IGA–UECM. The new inversion method of hydraulic conductivity and the proposed cement grouting curtain layout in this study offer possible strategies for the design, construction, and management of concrete-face rockfill dams.

## 1. Introduction

A concrete-face rockfill dam has the advantages of low cost, strong adaptability to complex terrain and environment, and suitable anti–seepage performance [1]. It has become a mainstream type of high dam worldwide [2,3]. Analyzing seepage is crucial to hydraulic engineering, leading to approximately 25% of dam failures worldwide [4]. Therefore, the seepage stability analysis of a concrete rockfill dam, which requires hydraulic conductivity, is critical to its design, construction, and management. However, the accurate hydraulic conductivity of a dam’s foundation needs to be inverted because a geological exploration can only determine the hydraulic conductivity range of different layers of the foundation and several borehole water heads.

Numerical algorithms are the most widely used method for parameter inversion in hydraulic engineering, but the accuracy of numerical inversion methods wildly used in engineering sometimes cannot meet design and construction needs [5]. For example, the Bayes [6,7], Kalman [8,9], and Gioda [10] numerical algorithms utilize the experience of engineering technicians or the back analysis results of several previous groups and are most widely used in hydraulic engineering [11]. However, the uncertainty of geological conditions and field measurement errors significantly impact the inversion results. The inversion of hydraulic conductivity can be transformed into an optimization problem by designing an appropriate objective function with a mathematical seepage model [12,13]. Deep learning methods can effectively improve computational efficiency and accuracy of optimization problems. At present, genetic algorithm (GA) [14], Bayesian optimization algorithm (BO) [15], and tree-structured Parzen estimator (TPE) [16] are the research hotspots of optimization technology based on deep learning. However, using deep learning to improve the accuracy and efficiency of hydraulic conductivity inversion is still challenging because these methods need to be improved based on a full consideration of engineering practicability.

The challenge of deep learning research is the hyperparameter selection, which reflects the learning rate [17,18,19,20]. GA transforms the problem-solving process into a process similar to the crossover and mutation of chromosome genes in biological evolution through mathematical means and computer simulation, which are advantageous in solving complex combinatorial optimization problems [21,22]. However, premature convergence often occurs in a GA calculation and only returns the local optimal solution rather than the global one [23,24,25,26]. GA in hydraulic conductivity inversion must be improved according to the characteristics of the three-dimensional seepage field mathematical model of a dam. BO can absorb the experience of previous hyperparameters and use a Gaussian model [27] to select the subsequent hyperparameter combination more quickly and efficiently [28]. TPE is similar to BO but uses a Gaussian mixture model [29] to learn the hyperparametric model. These methods have their advantages in solving optimization problems, but their suitability for inversion analysis of the three-dimensional seepage field of a dam needs further study.

In addition to optimization models, the rules of an inverted seepage field and the accuracy of hydraulic conductivity are greatly affected by mathematical seepage models [30,31,32]. Currently, the most widely used seepage model for calculating fractured rock mass is the discontinuous medium seepage model (DMSM) [33], which ignores the permeability of a rock block so that only seepage occurring in the fracture [34,35,36] is close to the actual flow in a fractured rock mass. However, many fracture parameters, such as fracture width, length, and filling condition, are required to establish the model and are challenging to obtain through geological exploration, leading to a low application in engineering. Another popular seepage model of fractured rock mass is the equivalent continuum model (ECM), which uses the permeability tensor to reflect the permeability of fractured rock mass, and seepage can exist at any point in a region [37,38,39]. Although the flow movement assumed by the ECM model is slightly different from the actual flow, the permeability tensor of the ECM can be obtained from the statistical parameters of the fracture distribution characteristics in a geological exploration and has a greater application in engineering. Because of the constant water level change, many dam parts are usually unsaturated; thus, the unsaturated equivalent continuum model (UECM) [40] is more suitable for dams. The current research on the ECM and UECM mainly focuses on fractured rock mass because laboratory seepage test data can support them [37,38,39,40]. However, a dam’s size is much larger than that of the fractured rock mass, and the influence of dam structures and geology should also be considered. The assumption of the UECM, the establishment of a dam and its foundation model, and the code of the UECM require modification using a large number of engineering data to meet the engineering requirements. In particular, some dams are still in the design or construction stage without water storage and lack hydraulic monitoring data, which requires an accurate and universal calculation code. Therefore, it is necessary to study a new UECM code to calculate different dam cases to meet the needs of water conservancy facilities in various regions. In addition, there are few inversion methods coupled with the UECM.

The literature review shows that existing inversion methods of dam hydraulic conductivity are either not accurate enough or too complex to be directly used in engineering. Therefore, this paper proposes a new method (IGA–UECM) for the inversion of dam foundation hydraulic conductivity with a high engineering applicability, which is realized by improving the standard GA and coupling with the UECM. The IGA–UECM has the advantage of avoiding premature and fast calculations. The validity of this method is verified by a geological survey and design data of the Hami concrete-face rockfill dam in China. The major contributions of this study are as follows:

1. The standard GA algorithm is improved by creating a new genetic operation to overcome the premature convergence shortage of the standard GA in the hydraulic conductivity inversion problem. The improved genetic algorithm (IGA) has a faster convergence speed than BO and TEP on hydraulic conductivity inversion.
2. A new method and code for the inversion of dam foundation hydraulic conductivity by coupling the improved GA and the UECM is proposed, which fully considers engineering practicability. The realization of the three-dimensional finite element solution of the UECM is based on our previous research.
3. The geological survey and design data of the Hami concrete-face rockfill dam are used to verify the new method for the inversion of dam foundation hydraulic conductivity, and an engineering application case of the new method is presented.
4. Some suggestions are given for the inversion dam foundation hydraulic conductivity, the three-dimensional seepage field calculation, and the anti-seepage curtain layout of a concrete-face rockfill dam.

## 2. Method

First, the three-dimensional finite element mesh models of the dam in the natural period and the operation period were established according to the topographic map and section structure map. Then, the dam foundation’s hydraulic conductivity was inversely analyzed by a deep-learning optimization algorithm coupled with the UECM according to the water head of the drilling exploration boreholes in the natural period to obtain an accurate value. Next, the validity of the inversion method is verified by the geological survey and design data of the dam.

### 2.1. An Improved Genetic Algorithm for Inversion of Dam Foundation’s Hydraulic Conductivity

**Figure 1:**
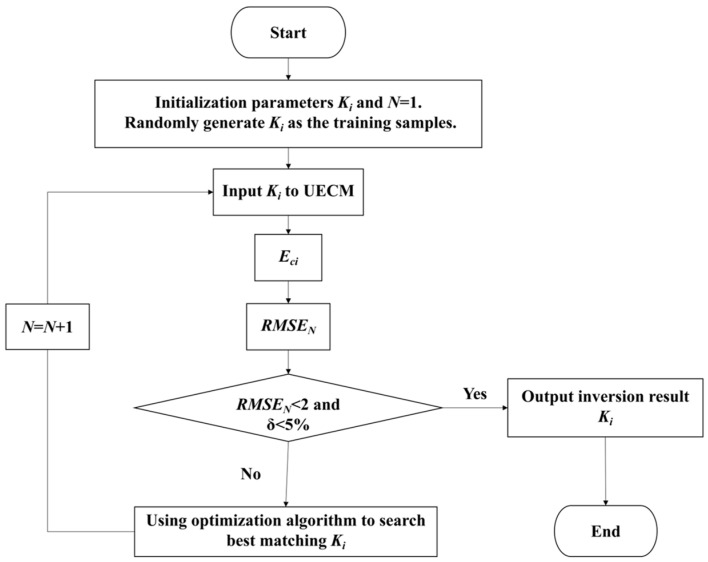
Flowchart for the inversion of the Hami concrete-face rockfill dam foundation’s hydraulic conductivity.

The inversion of a dam foundation’s hydraulic conductivity is meant to find the optimal solution of the objective function by using deep learning methods and the drilling water head to reverse the hydraulic conductivity. According to the design criteria of hydraulic engineering, the relative error between the calculated water head from inversion and the drilling water head should be less than 5%. We used mean squared error (MSE) [41] to construct an objective function, which reflected the samples’ fluctuation. We used the calculated relative error of the water head (*δ*) as the constraint condition. The smaller the MSE and *δ* (the smaller the deviation between the calculated water head and the drilling water head), the better the inversion. Considering the influence of the foundation’s hydraulic conductivity on the seepage field in hydraulic engineering, we defined the *MSE_N_* as less than 2 and *δ* as less than 5% as the iteration ended. The flow field diagram of the inversion analysis of the hydraulic conductivity of the dam foundation is shown in Figure 1.

(1) $$MSE_{N}=\frac{1}{n}\overset{n}{\underset{i=1}{\sum }}{\left(E_{mi}-E_{ci}\right)}^{2}$$

(2) $$\delta =\left|\frac{E_{ci}-E_{mi}}{H_{max}-H_{min}}\right|\%$$

where *E_mi_* is the drilling water head at point *i*; *E_ci_* is the calculated water head at point *i*; n is the number of drilling boreholes; *N* is the number of iterations; *H_max_* is the highest groundwater level; and *H_min_* is the lowest groundwater level.

**Figure 2:**
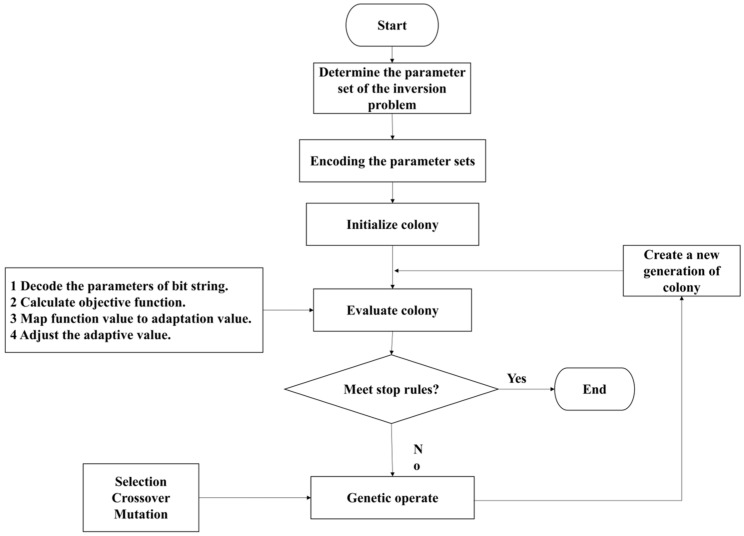
The flow chart of the genetic algorithm.

**Figure 3:**
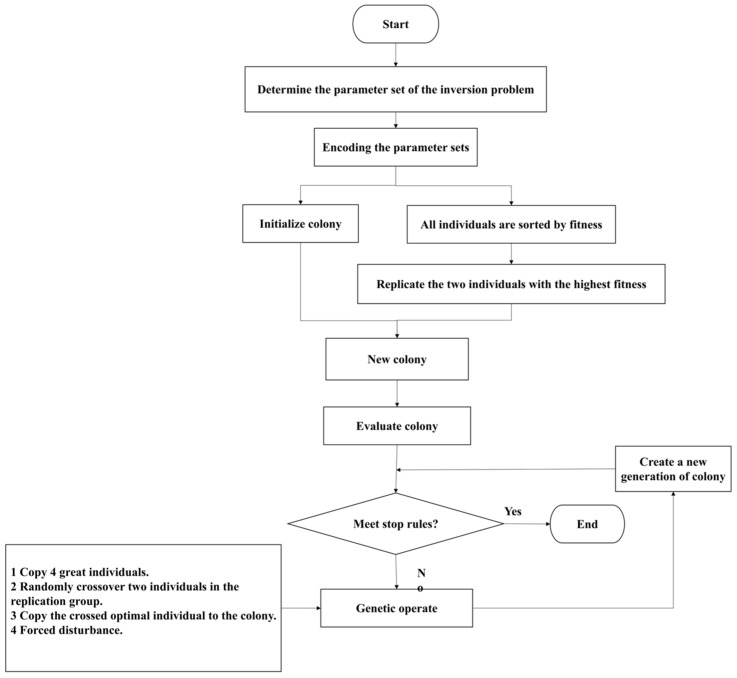
The flowchart of the improved genetic algorithm.

GA [21] is an algorithm that simulates the biological evolution mechanism in nature. It regards the parameter set as a population in the biological world and sets the optimal parameter by selecting the best one through survival. In a genetic algorithm, after the initial population is formed by coding, the individuals of the population are evaluated to perform certain operations according to their adaptability to the environment to achieve continuous optimization, thus approaching the optimal solution. Genetic operation includes three genetic operators, which are selection, crossover, and mutation. Selection refers to selecting superior individuals from the population to eliminate inferior ones. It aims to directly transfer the optimized individuals to the next generation or to generate new individuals through pairing and crossover to the next generation. Crossover refers to the replacement and reorganization of part of the structure of two husband generation individuals to generate new individuals so that leaps and bounds improve the searchability of the GA. Mutation refers to changing some gene values of individual strings in a population. It allows the GA to search locally to accelerate the convergence to an optimal solution while maintaining population diversity to prevent premature convergence. The effect of the genetic operation is related to the operation probability, coding method, population size, initial population, and setting of the fitness function. The operation of an individual genetic operator is carried out under random disturbance, so the rule of individual migration to the optimal solution in the population is random. The genetic operation of a GA is a directed search rather than a directed search of the traditional random search method, making it more efficient. The processes of the GA are as follows (Figure 2):

However, we found premature convergence when we used the GA to solve Equations (1) and (2). Therefore, according to the characteristics of the dam foundation’s hydraulic conductivity inversion, we proposed a new, improved GA algorithm (IGA) and established a program called IGA–Shen for inversion using the IGA coupled with the UECM. In the early stage of the GA, a super individual appears in the population, and the fitness of this individual greatly exceeds the average individual fitness of the current population. As a result, the individual soon occupies an absolute proportion of the population, the population’s diversity rapidly decreases, and the evolutionary ability of the population is lost so that the algorithm converges to the local optimal solution earlier. We improved the GA to eliminate this shortage, and the flow chart is shown in Figure 3. In the initial population, all individuals are sorted according to their fitness, and then the support and confidence of these individuals are calculated. The two individual structures with the highest fitness in the current population are thoroughly copied into the mating population. Four copies are made according to the principle of good individuals, and no copies are made for poor individuals. We randomly selected two individuals from the replication group, crossed the two individuals many times, and selected the best individual from the results to store in the new population. In addition, we also disturbed the population of each generation in a small range to maintain the population diversity. While adopting the optimal retention strategy, the best individual was forced to perform a small range of mutation operations. The specific scope was affected by the grid number, parameter number, and parameter variation range of the UECM, so it was necessary to carry out a trial calculation for different models.

### 2.2. Seepage Field Calculation by the Unsaturated Equivalent Continuum Model

We used FORTRAN to compile a program (Shen–HHU) to calculate the dam’s three-dimensional seepage field using the UECM. The Shen–HHU had been verified in previous studies [42,43,44].

#### 2.2.1. Basic Differential Model

Soil is composed of solid, liquid, and gas phases, and its permeability is determined by the properties of these three phases and their interactions. The hydraulic conductivity of unsaturated soil is a function of soil saturation [40]:

(3) $$\frac{\partial }{\partial x_{i}}\left[k_{ij}^{s}k_{r}\left(h_{c}\right)\frac{\partial h_{c}}{\partial x_{j}}+k_{i3}^{s}k_{r}\left(h_{c}\right)\right]-Q=\left[C\left(h_{c}+\beta S_{s}\right)\right]\frac{\partial h_{c}}{\partial t}$$

where *h_c_* is the water pressure head; $k_{ij}^{s}$ is the saturated hydraulic conductivity tensor; *k_i_*_3_ is the hydraulic conductivity value only related to the third coordinate axis in the saturated hydraulic conductivity tensor; *h_r_* is the relative water permeability, which is 0 < *h_r_* < 1 in the unsaturated area and 1 in the saturated area; *C* is the specific water capacity; *β* is a constant, which is 0 in the unsaturated area and 1 in the saturated area; *S_s_* is the elastic specific storage; and *Q* is the source–sink term.

#### 2.2.2. Definite Solution Condition

The effect of rainfall on soil permeability should be considered when solving Equation (3). Therefore, the initial condition of Equation (3) is as follows [40]:

(4) $$h_{c}\left(x_{i},t\right)=h_{c}\left(x_{i},t_{0}\right)$$

Furthermore, the boundary condition of Equation (3) is as follows [40]:

(5) $$\left\{\begin{array}{c} h_{c}\left(x_{i},t\right)\left|\Gamma_{1}\right.=h_{c1}\left(x_{i},t\right)\\ -\left[k_{ij}^{s}k_{r}\left(h_{c}\right)\frac{\partial h_{c}}{\partial x_{j}}+k_{i3}^{s}k_{r}\left(h_{c}\right)\right]n_{i}\left|\Gamma_{2}\right.=q_{n}\\ -\left[k_{ij}^{s}k_{r}\left(h_{c}\right)\frac{\partial h_{c}}{\partial x_{j}}+k_{i3}^{s}k_{r}\left(h_{c}\right)\right]n_{i}\left|\Gamma_{3}\right.\geq 0\;\;h_{c}\left|\Gamma_{3}\right.=0\\ -\left[k_{ij}^{s}k_{r}\left(h_{c}\right)\frac{\partial h_{c}}{\partial x_{j}}+k_{i3}^{s}k_{r}\left(h_{c}\right)\right]n_{i}\left|\Gamma_{4}\right.=q_{r}\left(t\right) \end{array}\right.$$

where *n_i_* is the cosine of normal direction outside the boundary plane; *t*_0_ is the initial time; *h_c_*_1_ is the known water head; *q_n_* is the known seepage discharge; *q_r_*(*t*) is the rainfall infiltration seepage discharge; *h_c_*(*t*_0_) is the seepage field water head at the initial time; $\Gamma_{1}$ is the known water head boundary; $\Gamma_{2}$ is the known seepage discharge boundary; $\Gamma_{3}$ is the rainfall infiltration boundary; and $\Gamma_{4}$ is the saturated escape surface boundary.

#### 2.2.3. Solution Method

The numerical solution of Equation (3) was calculated using domain discretization. Domain discretization uses finite discrete points to replace the original continuous space. The specific process is as follows: divide the calculated area into many complementary and overlapping sub-areas, and determine each sub-area’s node position and the node’s control volume. A node is the minimum geometric unit of the geometric position, control volume, application of control equation, or conservation theorem of the unknown physical quantities to be solved. Generally, nodes are regarded as representatives of the control volume. At the beginning of region discretization, the small regions divided by a series of lines or curve clusters corresponding to the coordinate axis are called the sub-regions. The control volume and sub-regions do not always coincide. The grid is the basis of discretization, and its nodes are the storage locations of discretized physical quantities. Commonly used discretization methods are the finite difference method, the finite volume method, and the finite element method. Commercial CFD software, such as FLUENT2021.R1 and CFX2021.R1, uses the finite volume method. However, the finite element method (FEM) can formulate methods for different order basis functions and has higher grid accuracy and flexibility. Considering the complexity of the unsaturated continuum equation and the mathematical model of practical engineering, the Shen–HHU uses the finite element method to solve Equations (1)–(3). The specific derivation process of Equations (6)–(20) is shown in the previous research of the corresponding author [45], and this section only quotes the main formula.

We discretized the computational spatial domain into finite elements, such as *N* elements. For each element (8—node hexahedral isoparametric element), we selected the appropriate shape function *N_m_*(*x_i_*) to satisfy the following:

(6) $$h_{c}\left(x_{i},t\right)=N_{m}\left(x_{i}\right)h_{cm}\left(t\right)\;\;i=1,2,3$$

where *N_m_*(*x_i_*) is the unit shape function; *h_cm_*(*t*) is the unit node water pressure head; and *h* is the total water head, which is *h* = *h_c_* + *x*_3_.

We substituted Equation (6) with Equation (3) to calculate the residual error as follows:

(7) $$R=\overset{3}{\underset{i=1}{\sum }}\overset{3}{\underset{j=1}{\sum }}\frac{\partial }{\partial x_{i}}\left[k_{r}\left(h\right)k_{ij}\frac{\partial }{\partial x_{i}}\left(N_{m}h_{cm}+x_{3}\right)\right]-\left[C\left(h\right)+\beta S_{s}\right]\frac{\partial }{\partial t}\left(N_{m}h_{cm}\right)-Q$$

We applied the Galerkin weighted residual method [46], making *N_m_*(*x_i_*) a weight function (*W_m_*(*x_i_*) = *N_m_*(*x_i_*)). So, for the function *h_c_*(*x_i_*, *t*) to approach the exact solution of the partial differential equation, it is necessary to satisfy the Equation (8) calculation area *G*.

(8) $${∭}_{G}RWdG={∭}_{G}\left\{\overset{3}{\underset{i=1}{\sum }}\overset{3}{\underset{j=1}{\sum }}\frac{\partial }{\partial x_{i}}\left[k_{r}\left(h\right)k_{ij}\frac{\partial }{\partial x_{j}}\left(N_{m}h_{cm}+x_{3}\right)\right]\right.\left.-\left[C\left(h\right)+\beta S_{s}\right]\frac{\partial }{\partial t}\left(N_{m}h_{cm}\right)-Q\right\}N_{n}dG=0$$

Using Green’s first formula [47] for Equation (8), we calculated the following:

(9) $$\begin{array}{l} \iint_{G}\overset{3}{\underset{i=1}{\sum }}\overset{3}{\underset{j-1}{\sum }}k_{r}\left(h\right)k_{ij}\frac{\partial N_{n}}{\partial x_{i}}\frac{\partial }{\partial x_{j}}\left(N_{m}h_{cm}\right)dG+\iint_{G}\overset{3}{\underset{i=1}{\sum }}k_{r}\left(h\right)k_{i3}\frac{\partial N_{n}}{\partial x_{i}}dG\\ ={∯}_{S}N_{n}k_{r}\left(h\right)\overset{3}{\underset{i=1}{\sum }}\left[\overset{3}{\underset{j=1}{\sum }}k_{ij}\frac{\partial }{\partial x_{j}}\left(N_{m}h_{cm}\right)+k_{i3}\right]n_{i}dS-{∭}_{G}\left[C\left(h\right)+\beta S_{s}\right]N_{n}\frac{\partial }{\partial t}\left(N_{m}h_{cm}\right)dG-{∭}_{G}SN_{n}dG \end{array}$$

where *S* is the boundary of the computational domain.

For the whole discrete calculation domain,

(10) $$\begin{array}{l} \overset{NE}{\underset{e=1}{\sum }}\left[{∭}_{G_{e}}^{}k_{r}^{e}k_{ij}^{e}\frac{\partial N_{n}^{e}}{\partial x_{i}}\frac{\partial }{\partial x_{j}}\left(N_{m}^{e}h_{cm}\right)dG\right]\\ =\overset{NE}{\underset{e=1}{\sum }}\left[{∯}_{S_{e}}^{}N_{n}^{e}k_{r}^{e}\left(h\right)\overset{3}{\underset{i=1}{\sum }}\left[k_{ij}^{e}\frac{\partial }{\partial x_{j}}\left(N_{m}^{e}h_{cm}\right)+k_{i3}^{e}\right]n_{i}dS-{∭}_{G_{e}}^{}\overset{3}{\underset{i=1}{\sum }}k_{r}^{e}\left(h\right)k_{i3}^{e}\frac{\partial N_{n}^{e}}{\partial x_{j}}dG-{∭}_{G_{e}}^{}SN_{n}^{e}dG\right] \end{array}$$

where the symbol with “*e*” indicates the quantity corresponding to the unit.

The governing equation of the unit is as follows:

(11) $${\left[K\right]}^{e}{\left\{h_{c}\right\}}^{e}+\left({\left[S\right]}^{e}\right){\left\{\frac{\partial h_{c}}{\partial t}\right\}}^{e}={\left\{F\right\}}^{e}$$

(12) $$K_{ab}^{e}=\int_{\Omega^{e}}K_{r}\left(h_{c}\right)\left(N_{a,i}K_{ij}^{s}N_{b,j}\right)d\Omega$$

(13) $$S_{ab}^{e}=\int_{\Omega^{e}}^{}\left[C\left(h_{c}+\beta S_{s}\right)\right]N_{a}N_{b}d\Omega$$

(14) $$F_{\left(a\right)}^{e}=-\int_{\Omega^{e}}K_{r}\left(h_{c}\right)\left(N_{a,i}K_{ij}^{s}Z_{,j}\right)d\Omega +\int_{\Gamma_{2}}qN_{a}d\Gamma$$

where *a* = 1, 2, …, 8; *b* = 1, 2, …, 8; *I* = 1, 2, 3; *j* = 1, 2, 3; *N_a_* and *N_b_* is the unit shape function; and *h_c_* is the water pressure head. We integrated the element governing equations to obtain the global finite element governing equation as follows:

(15) $$\left[K\right]\left\{h_{c}\right\}+\left[S\right]\left\{\frac{\partial h_{c}}{\partial t}\right\}=\left\{F\right\}$$

We adopted an implicit finite difference scheme for time ($\frac{\partial h_{c}}{\partial t}=\frac{1}{\Delta t}\left[{\left(h_{c}\right)}_{t+\Delta t}-{\left(h_{c}\right)}_{t}\right]$) and brought it into Equation (15) to obtain the solution:

(16) $$\left(\left[K\right]+\frac{1}{\Delta t}\left[S\right]\right){\left\{h_{c}\right\}}_{t+\Delta t}=\left\{F\right\}+\frac{1}{\Delta t}\left[S\right]{\left\{h_{c}\right\}}_{t}$$

#### 2.2.4. Iterative Format

We used the incremental iteration method [48] and defined ${\left\{h_{c}\right\}}_{t+\Delta t}^{k+1}={\left\{h_{c}\right\}}_{t+\Delta t}^{k}+{\left\{\Delta h_{c}\right\}}_{t+\Delta t}^{k+1}$ to derive the following iterative format suitable for calculation:

(17) $$\left[A\right]{\left\{\Delta h_{c}\right\}}_{t+\Delta t}^{k+1}={\left\{\Delta B\right\}}_{t+\Delta t}^{k+1}$$

among which,

(18) $$\left[A\right]=\left[K\right]+\frac{1}{\Delta t}\left[S\right]$$

(19) $${\left\{\Delta B\right\}}_{t+\Delta t}^{k+1}={\left\{\Delta F\right\}}_{t+\Delta t}^{k}-\frac{1}{\Delta t}\left[S\right]\left({\left\{h_{c}\right\}}_{t+\Delta t}^{k}-{\left\{h_{c}\right\}}_{t}\right)$$

(20) $${\left\{\Delta F\right\}}_{t+\Delta t}^{k}=-\int_{\Omega^{e}}K_{r}\left(h_{c}\right)\left(N_{a,i}K_{ij}{\left({\left(h_{c}\right)}_{t+\Delta t}^{k}+x_{3}\right)}_{,j}\right)d\Omega +\int_{\Gamma_{2}}qN_{a}d\Gamma$$

We used the eight-node hexahedron isoparametric element to calculate the seepage pressure field according to Equations (11)–(20).

#### 2.2.5. Seepage Discharge

The seepage discharge refers to the amount of water passing through the cross section of porous media perpendicular to the seepage flow direction in unit time, which is calculated as follows [49]:

(21) $$q=\sum \overset{}{\underset{\Delta }{\iint }}K\frac{\partial H}{\partial n}{\mathrm{dS}}_{n}$$

where Δ denotes a specified cross section, and {*H*} denotes the hydraulic conductivity of the specified cross section in the normal external direction for the hydraulic gradient [21,41].

### 2.3. Other Deep Learning Methods

BO [28] is based on the Bayesian posterior distribution theory that preliminarily iterates the global minimum of the cluster objective function. It does not require many random searches like the random cluster algorithm and has the advantages of good robustness and fast convergence. According to the preliminary information of the objective function, the algorithm can quickly find the following optimal location in the parameter space under the current known information and add the optimal location information to the subsequent iterations to achieve the optimal solution as soon as possible.

TPE uses GMM to learn the hyperparameter. After introducing Bayes to the inversion problem, *p*(*x*|*y*) is the conditional probability that the hyperparameter is *x* when the model loss is *y*. A loss threshold y* is selected according to existing data, and two probability densities *l*(*x*) and *g*(*x*) are learned, respectively, for the data that are greater than the threshold and less than the threshold. A new x* according to *g*(*x*)/*l*(*x*) is determined, and *g*(*x*)/*l*(*x*) is continuously minimized until the preset time is reached. The equation of *p*(*x*|*y*) is as follows [16]:

(22) $$p\left(x|y\right)=\left\{\begin{array}{c} l\left(x\right)\;\;if\;y<y^{*}\\ g\left(x\right)\;\;\;if\;y\geq y^{*} \end{array}\right.$$

where *l*(*x*) is the density formed using the observations {*x*^(*i*)^} such that the corresponding loss *f*(*x*^(*i*)^) is less than y*, and *g*(*x*) is the density formed by using the remaining observations.

## 3. Engineering Example

### 3.1. Project Overview and Data Collection

**Figure 4:**
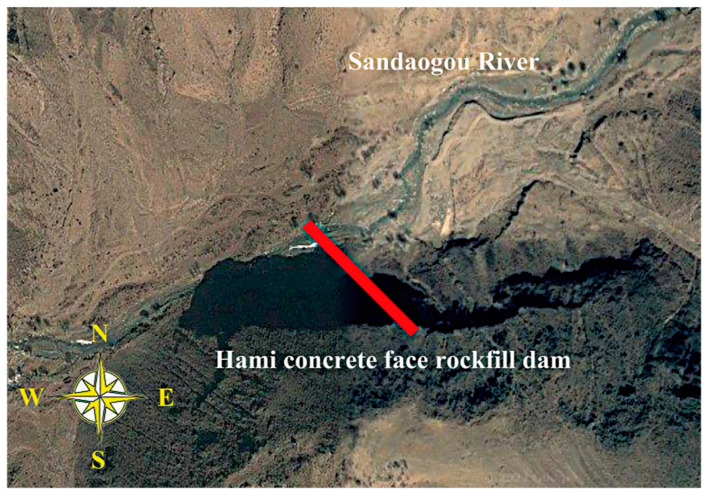
Location of the Hami concrete-face rockfill dam.

The Hami Pumped Storage Power Station under construction is a pure pumped storage power station located northeast of Hami City, Xinjiang Province, China. Its lower reservoir concrete-face rockfill dam is located on the Sandaogou River (Figure 4). Groundwater in the lower reservoir area mainly includes Quaternary pore phreatic water and bedrock fissure water. Abundant pore phreatic water is stored in the riverbed with strong water permeability at the bottom of the valley and in the pores of colluvial and eluvial rubble layers at the foot of the slopes on both banks. The pore phreatic water is recharged by upstream river water, precipitation, and a small amount of groundwater, and it is discharged into the Sandaogou River. There is no groundwater outcrop on the surface of either bank. The permeability of bedrock in the dam site area is mainly affected and controlled by lithology, rock integrity, fracture development, bank slope unloading, and rock weathering. According to the water pressure test results of 12 boreholes in 95 sections at the dam site, 85 sections with weak permeability accounted for 95.2%, and 10 sections with slight permeability accounted for 4.76%. This project’s foundation rock mass was measured by using a drilling water pressure test in the geological exploration and labeled according to the permeable rate range (the unit is Lu) in Table 1. Generally, geological exploration can only determine the experience range of its hydraulic conductivity according to the classification of rock mass and the range of permeable rate. The exact value of the hydraulic conductivity must be obtained by an inversion analysis of the borehole water head.

**Figure 5:**
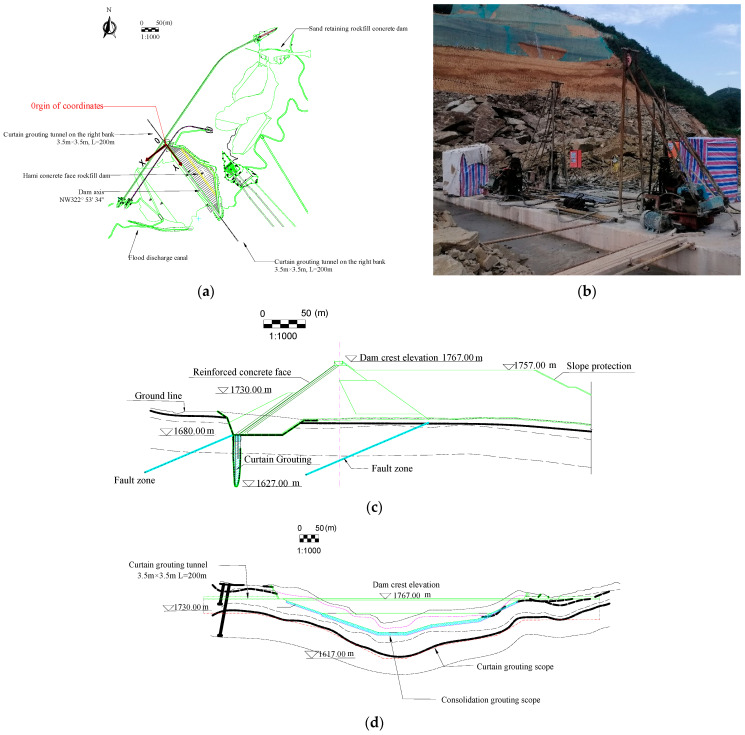

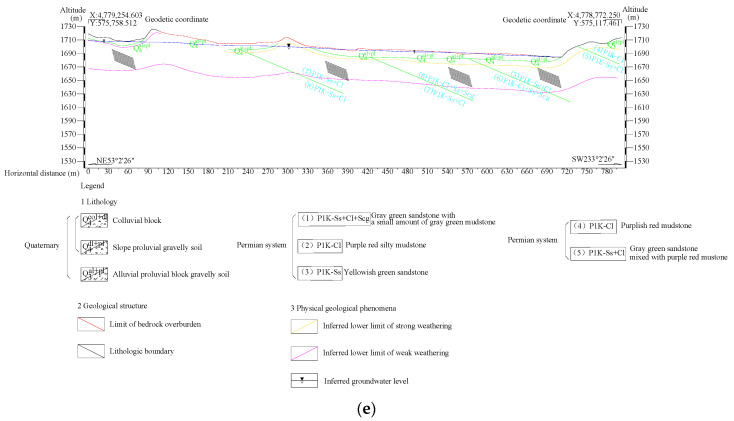
Hami concrete-face rockfill dam schematic diagram. (**a**) The vertical view. (**b**) Photos of the grouting curtain construction site. (**c**) The standard sectional view. (**d**) The view of the dam unfolding along the dam axis. The coordinate origin of the simulation model is marked in red. (**e**) Geological structure of the standard sectional view.

The normal water level of the lower reservoir is 1761.0 m a.s.l; the dead water level is 1732.0 m a.s.l; the flood water level is 1761.5 m a.s.l; the check flood level is 1761.6 m a.s.l; and the regulation capacity is 7.22 million m^3^. The foundation of the riverbed toe slab of the concrete-face rockfill dam is built on weakly weathered bedrock to fresh bedrock. The minimum elevation of the toe slab foundation surface is 1680.0 m a.s.l; the dam crest elevation is 1767.0 m a.s.l; the dam height is 87.0 m a.s.l; and the dam crest length is 565.2 m. The dam crest is 10.0 m wide. A 6.2 m height “L” wave wall stands upstream of the dam crest. The top wall elevation is 1768.2 m a.s.l, and the bottom elevation is 1762.0 m a.s.l, which is 1 m higher than the normal water level. The upstream dam slope is 1:1.4, the downstream dam slope is 1:1.4, and the comprehensive slope ratio is 1:1.66. A random backfill area is set behind the dam. The elevation of the platform top is 1763.0 m a.s.l, and the downstream slope is 1:2.0. The anti-seepage system of the concrete-face rockfill dam includes a concrete-face slab, a toe slab, and a cement grouting curtain. Curtain grouting is a process in which cement grout is injected into the cracks and pores of the rock mass or soil layer to form a continuous water-blocking curtain to reduce seepage flow and pressure, and it is the primary anti-seepage treatment of hydraulic building foundations. The grouting curtain is 1045.0 m long along the dam axis, with an average depth of 53.0 m and a thickness of 8.0 m. Figure 5 depicts the expansion and standard section diagrams of the Hami concrete-face rockfill dam along the dam’s toe slab line.

The calculation scope of the three-dimensional finite element model of the natural period of the dam should include all the positive measurement points of the drilling water pressure near the dam in the geological exploration. The calculation range of the three-dimensional finite element model during the dam operation period should be more than twice the dam height (174 m). The model scope of the Hami dam in the operation period was extended to cover its upstream sand-retaining dam, which was larger than the calculation area in the natural period. In addition, since the fault near the dam greatly influenced its seepage field, the calculation scope of the model was appropriately extended to the fault area nearby. The model above the groundwater level in the extended part was simplified because the terrain above the groundwater level did not affect the seepage field near the dam. The model’s coordinate origin and axis direction are shown in Figure 5a. Its earth geodetic coordinates are (x, y) = (575,654.299, 4779,524.6173). In the coordinate system of our model, the *X* axis is along the river, which is perpendicular to the dam axis, and the direction pointing downstream is positive; the *Y* axis is along the dam axis, and it is positive when it points to the left bank; and the *Z* axis is vertical, taking the elevation as the coordinate. The upstream and downstream boundaries are, respectively, intercepted at 140 m above the upstream slope toe of the lower reservoir barrage and 330 m below the downstream slope toe. The left and right bank boundaries are intercepted at 800 m to the left and 110 m to the right of the right dam abutment. The top elevation is based on the actual terrain, and the bottom elevation reaches 1500 m a.s.l. The area of the dam model in the natural period was –265 m < *x* < 350 m, –110 m < *y* < 675 m, and 1500 m < *z*. The area of the dam model in the operation period was –920 m < *x* < 540 m, –110 m < *y* < 1200 m, and 1500 m < *z*. Table 2 shows the natural period’s groundwater head (*E_M_*) of the Hami dam’s 12 drilling points in the geological exploration.

The inversion validity was verified by comparing the water heads from the drilling exploration and the inversion. Table 3 shows the grouting curtain layout of different working conditions to study whether the dam’s anti-seepage system can ensure seepage safety based on the inversion result. The normal water level of 1761.00 m and the dead water level of 1732.00 m were taken as the upstream water level, and the lowest water level of 1685.00 m was taken as the downstream water level. The hydraulic conductivity of the grouting curtain in the working condition was 2.5 times greater than that in the design scheme to simulate the poor construction quality of curtain grouting. The dam seepage stability analysis included determining the position of the phreatic line, the seepage flow, the average velocity, and the gradient.

### 3.2. Three-Dimensional Finite Element Mesh Model and Boundary Conditions of the Dam

The structural shape of all partitions of the Hami concrete-face rockfill dam is simple, so we divided its three-dimensional model into structured hexahedral grids to reduce the number of nodes and grids and to increase computational efficiency. The dam and foundation were divided into many large hexahedrons in advance according to the different permeability and structure, with adjacent hexahedrons sharing the same node. Because the linear interpolation method is more efficient in dividing such a large volume of hexahedrons, this paper used the Lagrange linear interpolation method [50]. We assumed a rectangular coordinate system *η*–*ζ*–*ξ* first. The position of the node is the function r (*x*, *y*, *z*) on the calculation surface concerning *x*, *y*, and *z*, and the r (*x*, *y*, *z*) of each point on the boundary of the known control region was used to determine the corresponding r (*x*, *y*, *z*) of each node in the control region. Therefore, the known values on the boundary could be interpolated to obtain each node’s value in the control area.

**Figure 6:**
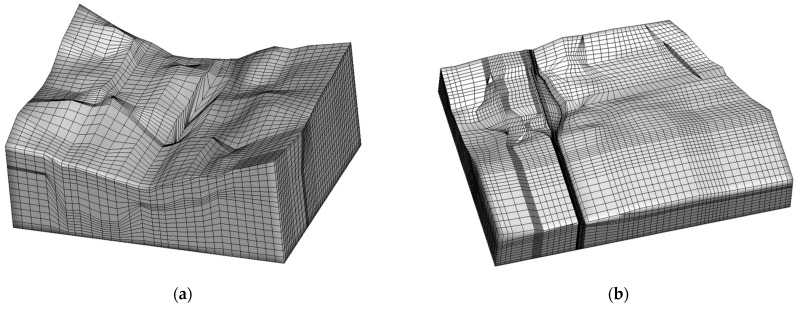
Three-dimensional finite element model of the Hami concrete-face rockfill dam. (**a**) Finite element model of the dam’s natural period. (**b**) Finite element model of the dam’s operation period.

Figure 6 shows the three-dimensional finite element mesh model of the Hami dam. Each dam structure’s grid is densified in the model. The total number of nodes of the dam’s three-dimensional finite element mesh model in the natural period is 35,234, and the number of elements is 32,136. There are 63,157 nodes and 58,019 elements in the dam’s three-dimensional finite element mesh model in the operation period.

The models have four boundary conditions: upstream boundary, downstream boundary, flow escape boundary, and impermeable boundary. The upstream boundary is the grid below the submergence line of the upstream water head in the dam site area. The grid of the downstream water level inundation line is the downstream boundary. The flow escape boundary includes the grid above the upstream and downstream water level inundation lines in the dam site area, the grid on the upstream faces of the left and right bank slopes, and all grids in contact with the atmosphere. The mesh at the bottom of the model belongs to the impermeable boundary.

### 3.3. Sensitivity Analysis on the Structure of the Mesh

We used the Shen-HHU to test the two models in Figure 6 to reduce the unit grid size by 10% and by 20% and to expand the unit grid size by 10% and by 20%. The result indicated that the relative error of the water level at the drilling points of the dam model in the natural period and in the operation period of the four cases was less than 0.1%. Because the relative error was too small, this paper did not use illustrations to explain. In addition, when the dam was in operation, its phreatic line position was basically unchanged with the change in unit grid size (the phreatic line position was the same as that in Figures 9 and 12 in Chapter 4). This indicated that the influence of mesh refinement on the calculation accuracy of the Shen-HHU could be ignored and that the mesh density in Figure 6 was reasonable. The size of the dam model in the natural period and in the operation period mainly depended on the height of the dam, the size of the grouting curtain, the location of the fault, and the depth of the impermeable stratum; the scope was large enough to allow us to ignore the impact of a slight change in size on the accuracy of the Shen-HHU. Therefore, we believe that grid processing is not a sensitive factor in the accuracy of the Shen-HHU.

## 4. Results and Discussion

### 4.1. Inversion Results of Dam Foundation Hydraulic Conductivity

#### 4.1.1. Inversion Results of IGA

**Figure 7:**
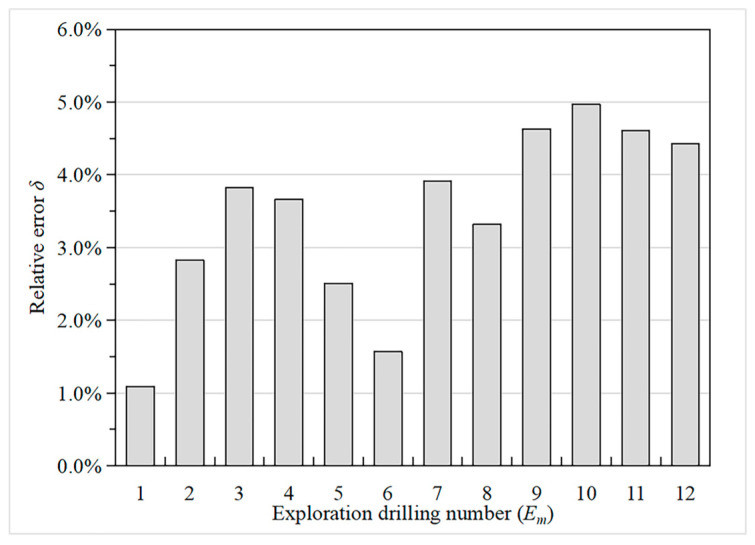
The relative error between the drilling water head and the calculated water head.

The hydraulic conductivity of the Hami dam foundation in the natural period inversed by the IGA is shown in Table 4. Figure 7 indicates that the relative errors between the calculated water head and the drilling water head of all drilling points are all less than 5%, which verify the validity of the IGA.

**Figure 8:**
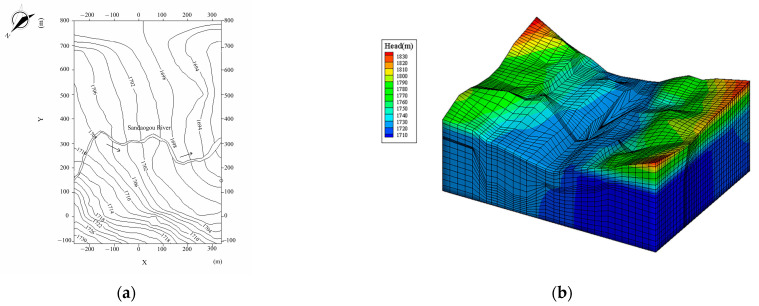
Results of the Hami concrete-face rockfill dam’s three-dimensional seepage field in the natural period. (**a**) Contour map of groundwater. (**b**) Three-dimensional seepage field.

**Figure 9:**
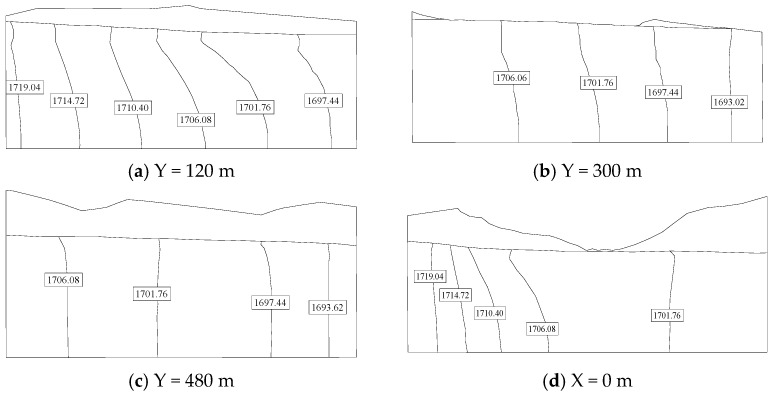
Groundwater potential map of the natural period profile of the Hami concrete-face rockfill dam. The unit of water level in the figure is in meter.

The contour map of the groundwater level in the natural period of the Hami dam calculated by the Shen–HHU and the potential distribution map of several typical profiles of the three-dimensional seepage field are shown in Figure 8 and Figure 9. Figure 8a shows that the isolines on the right bank are dense while the isolines on the left bank of the dam are sparse, indicating that the water level on the mountain on the right bank changes rapidly and the groundwater level on the left bank changes gently. The groundwater level of the mountain on the right bank is higher than on the left bank. There is a Kulai fault extending from the left bank of the dam body, where the isolines are relatively dense. The water level changes significantly, indicating a water-blocking effect. In addition, from the simulation results, the groundwater seepage gradient of the left dam abutment is slight, while that of the right dam abutment is large. From the potential distribution map of the selected profile (Figure 9), it can be seen that the groundwater level generally decreases from the right bank to the left bank and from the upstream to the downstream. In addition, by observing the contour map of the dam’s axis direction (Y direction), the right bank’s equipotential line is higher than the left bank’s equipotential line, which indicates that the right bank groundwater level changes rapidly, with a sizeable forced drop and noticeable change. In Figure 7, it can be seen that the relative errors between the calculated water head and the drilling water head are all less than 5%. Therefore, the IGA inversion analysis, the rock layer hydraulic conductivity, and the boundary conditions used in the inversion are appropriate.

#### 4.1.2. Comparison of the Improved Genetic Algorithm and Other Algorithms for Inversion of the Hydraulic Conductivity of the Dam Foundation

**Figure 10:**
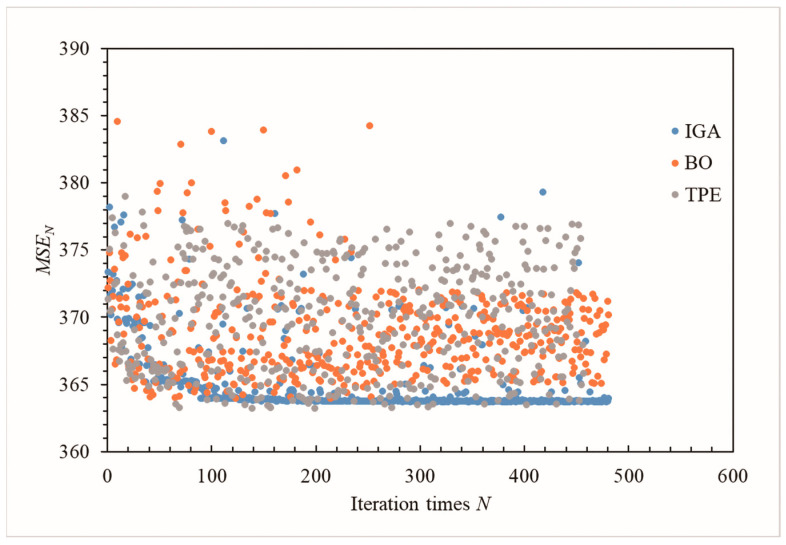
Comparison of the three algorithms for the inversion of the dam foundation’s hydraulic conductivity.

We used the IGA, the TEP algorithm, and the BO algorithm to invert the same model (Figure 6a and Equations (1)–(3)) to verify the superiority of the IGA in the inversion of the dam foundation’s hydraulic conductivity. Figure 10 illustrates that convergence starts when the IGA calculates the objective function for 100 iterations. However, the BO and the TPE algorithms still have not converged after 500 iterations of the objective function (Equations (1) and (2)), and their calculation efficiency is lower than that of the IGA. Because deep learning algorithms require good computer performance, we stopped after 500 iterations.

Some researchers believe that GA is limited in solving the optimal solution problem [51,52,53,54,55,56]. Many parameters of the three operators of a GA, such as crossover rate and mutation rate, can only be selected by experience, which affects the quality of the optimal solution. In addition, GA cannot use the network feedback information, leading to slow search speed, and it is prone to premature convergence when solving large-scale computing problems. When solving large-scale computing problems, BO and TPE algorithms are much more efficient than Gas because they can find better hyperparameter combinations with fewer steps [57]. However, when we used the IGA, the BO algorithm, and the TPE algorithm combined with the UECM to invert the same dam’s natural-period foundation hydraulic conductivity, we found that the IGA was more efficient than the others (Figure 10). We believe this is related to the engineering problem of inversion. Generally, the range of foundation hydraulic conductivity given by a geological exploration does not exceed 100 times, and the range of initial parameters is much smaller than that of optimization problems in other fields. In addition, the precise location of stratigraphic stratification is also given in a field survey. The number of foundation layers for dam construction does not exceed 10, and the hydraulic conductivity within 10 times can be simplified to the same material for calculation and analysis in hydraulic engineering. The advantages of the IGA are outstanding because of the small number of inversion parameters and the narrow range of the total number of samples. Our program Shen–HHU has high computational efficiency in solving seepage fields by using the UECM for the three-dimensional finite element model established in the natural period of the dam, which has been confirmed in previous studies. It results in the efficient inversion of foundation hydraulic conductivity by the IGA–Shen and the short calculation time. As long as the GA is improved appropriately to solve the premature defect, the inversion method of hydraulic conductivity suitable for a common three-dimensional finite element can be obtained. The IGA method is suitable only for the natural period model of the Hami concrete-face rockfill dam. There are many studies on improving GA [44,58,59,60,61,62,63,64,65,66,67]. The suitability of these improved methods for the inversion of a dam’s foundation hydraulic conductivity and their calculation efficiency need further study.

### 4.2. Influence of Different Layout Schemes of Dam Grouting Curtain on Dam Three-Dimensional Seepage Field

#### 4.2.1. The Three-Dimensional Seepage Field

**Figure 11:**
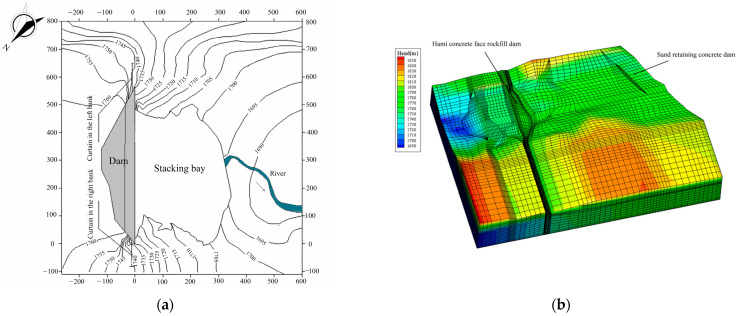
Results of the Hami concrete-face rockfill dam’s three-dimensional seepage field calculation. (**a**) Contour map of the groundwater level of the Hami concrete-face rockfill dam under HM-1. (**b**) Three-dimensional seepage field of the Hami concrete-face rockfill dam under HM-1.

**Figure 12:**
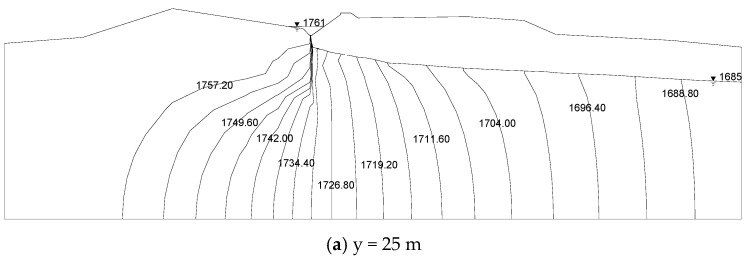

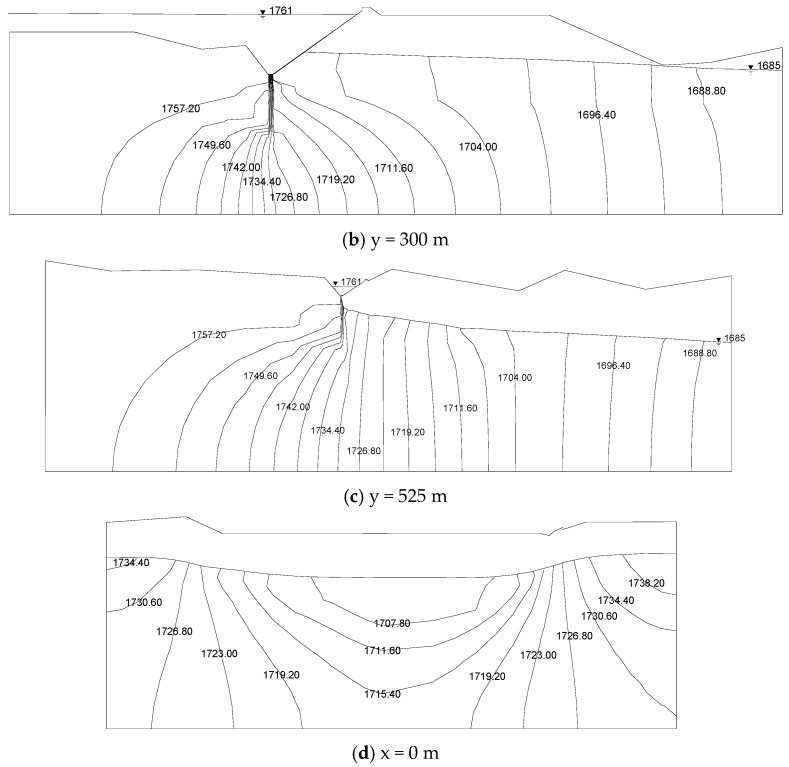
Groundwater potential map of the Hami concrete-face rockfill dam under HM-1. The unit of water level in the figure is in meter.

Figure 11 shows the contour map of the Hami concrete-face rockfill dam’s water level and the three-dimensional seepage field under the working condition HM-1. Figure 12 depicts the water head potential diagram of several typical sections under the working condition HM-1. Because of the limited space of the paper, we show only the three-dimensional flow field calculation results for the HM-1 condition. The laws of the seepage fields under other working conditions are similar. Figure 11a indicates that the reservoir water seeps downstream through the dam body, the grouting curtain on both banks, and the mountain. The soaking surface in the dam body downstream of the concrete face slab is relatively flat, showing a trend of being lower in the center of the riverbed and higher in the two dam abutments. The lowest soaking surface position is in the center of the riverbed. Because of the topographical asymmetry of the left and right banks, the seepage field in the dam site area is also asymmetric. The groundwater level on the right bank is higher than on the left. As the natural groundwater level in the reservoir area is low, there is noticeable seepage around the reservoir after impoundment. The reservoir water seeps downstream through the grouting curtain and the mountain. Figure 12 shows that the soaking surface forms a sudden drop at the upstream and downstream of the concrete face slab. In Figure 12, the place with the densest water level is the location of the grouting curtain.

#### 4.2.2. Suggestions on Optimization of Grouting Curtain

**Figure 13:**
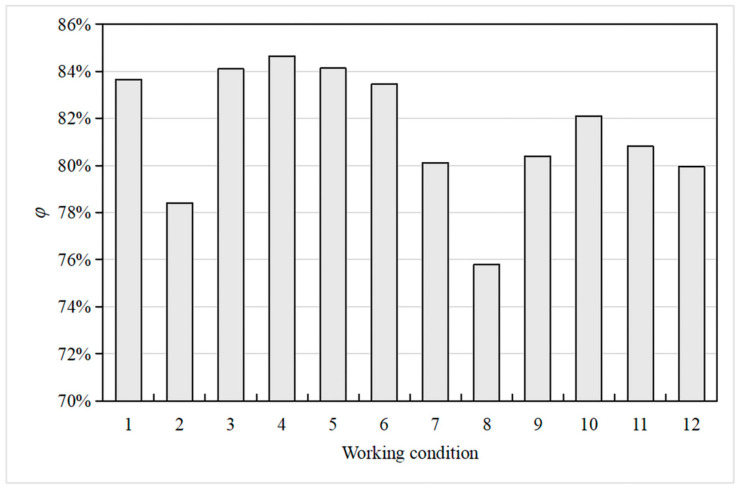
Water head reduction percentage of the anti-seepage system of the dam body and foundation.

The water head reduction percentage (*φ*) of the anti-seepage system of the dam body and foundation is shown in Equation (23). Figure 13 shows that *φ* of the design scheme of the Hami concrete-face rockfill dam is 83.64%, indicating that the anti-seepage effect of its anti-seepage system is significant. When the grouting curtain length on the left and right banks are all reduced by 20 m, which is 6.4% of the dam axis length (HM-6 and HM-12), *φ* is only reduced by 0.2%, which still meets the engineering requirements. However, when the curtain grouting construction quality is poor, *φ* is reduced by 5.24% under the HM-2 working condition and 4.32% under the HM-8 working condition. As the construction quality generally has little impact on the length and depth of the grouting curtain, the construction quality of curtain grouting is the most significant factor affecting the failure of seepage protection effect under the 12 working conditions. The permeability of a grouting curtain is generally tested using water pressure and acoustic geophysical tests at the construction site. During construction, each grouting curtain must be comprehensively tested to ensure the stability of the dam seepage. Under the same upstream water level, the overall deepening is better than the overall extension of the grouting curtain in water head reduction. When the overall depth of the grouting curtain is 20 m (HM-4 and HM-10), the grouting curtain and the panel reduce the water head by 64.33 m in total, and *φ* is 86.64%. However, compared to the scheme of extending the grouting curtain (HM-5 and HM-11), its (HM-4 and HM-10) effect on reducing *φ* is slight but requires more cement grouting by 1.5 × 10^5^ cm^3^. Therefore, increasing the length of the grouting curtain is more cost-effective in optimizing the anti-seepage effect. In conclusion, the anti-seepage effect of the system (faceplate and grouting curtain) of the dam body and dam foundation of the Hami dam is remarkable.

(23) $$\varphi =\frac{\left(H_{up}-H_{cu}\right)}{\left(H_{up}-H_{do}\right)}\times 100\%$$

where *H_up_* is the water level upstream of the dam; *H_do_* is the water level downstream of the dam; and *H_cu_* is the highest water level in the cushion.

**Figure 14:**
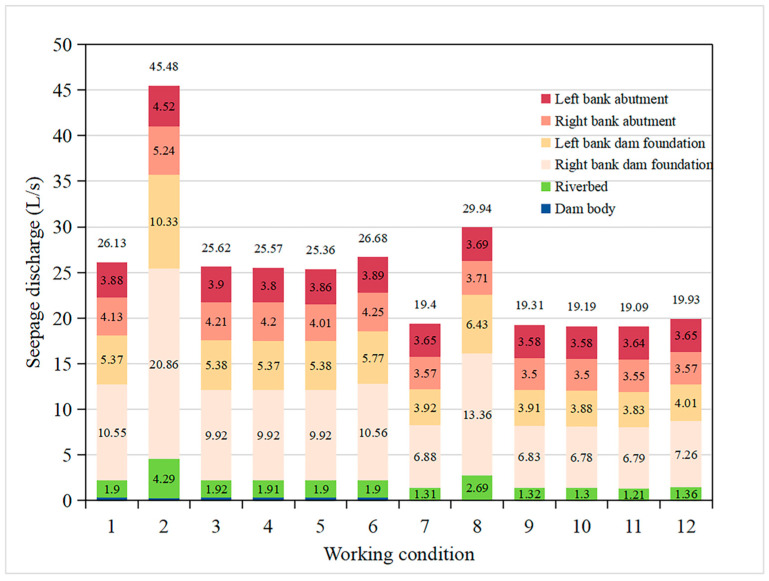
Seepage discharge of each partition of the Hami concrete-face rockfill dam’s axis section. The seepage discharges of the dam body are all less than 0.31 L/s, which is too small to be shown in the figure.

The maximum average seepage gradient of the dam grouting curtain under each working condition is shown in Table 5. Except for working conditions HM-3, HM-4, and HM-5, the maximum seepage gradient occurs at the grouting curtain under the dam body. The seepage gradient of the grouting curtain in all working conditions meets the design requirements. The most significant influence on the seepage gradient of the grouting curtain is the construction quality of the curtain grouting. However, if the hydraulic conductivity of the grouting curtain is not higher than 2.5 times the design value during grouting construction, the anti-seepage system can still ensure dam seepage stability. During construction management, special attention should be paid to the supervision and detection of the grouting construction quality of the grouting curtain under the dam foundation. Figure 14 shows the seepage discharge of each partition of the axis section of the Hami concrete-face rockfill dam. When the grouting curtain is under the HM-1 condition, the total seepage discharge of the dam is 26.13 L/s. If the construction quality of the curtain grouting is terrible (the hydraulic conductivity of the grouting curtain only increases 2.5 times), there is a sharp rise in the dam soaking surface, and the seepage flow increases by 74% (HM-2 and HM-7). The key factor that influences the seepage discharge of the dam is the construction quality of the curtain grouting. Deepening or extending the grouting curtain reduces the total seepage discharge slightly because the grouting curtain in the design scheme reaches the impermeable layer (three Lu). The difference in hydraulic conductivity between the impermeable layer and the grouting curtain is slight, resulting in a slight change in the dam’s total seepage discharge. Therefore, when selecting a dam site and designing the grouting curtain, it is better to extend the grouting curtain to the impermeable layer as far as possible to reduce the impact of the grouting curtain size on the seepage discharge of the dam.

The groundwater contour map, the potential map, the seepage gradient, and the seepage flow of the three-dimensional seepage of the Hami concrete-face rockfill dam under different grouting curtain layout schemes calculated by the IGA–UECM inversion results are reasonable. The regularity of the three-dimensional seepage field with different water levels and the same grouting curtain layout is consistent. This indicates that the IGA–UECM inversion method of dam foundation hydraulic conductivity is effective. Since the mathematical model of our algorithm inversion is a three-dimensional finite element grid, new parameters only need to be assigned to other structural elements. Our algorithm and code can be used for the hydraulic conductivity inversion of dam foundations and structures. The IGA can also be combined with other mechanical models to invert the mechanical parameters, except for the hydraulic conductivity.

## 5. Conclusions

The existing inversion methods of dam hydraulic conductivity are either not accurate enough or too complex to be directly used in engineering. Therefore, this paper proposes a new method of inverting a dam foundation’s hydraulic conductivity called the IGA–UECM. In order to overcome the premature convergence defect of GA, we proposed the IGA. Compared to the BO and TPE algorithms, the IGA has the advantage of fast convergence in the inversion of the dam foundation’s hydraulic conductivity. The seepage field of the Hami concrete-face rockfill dam with different grouting curtain arrangements in the operation period was calculated using the inversion results. The validity of the IGA–UECM was verified by considering the rationality of the inverted water head, the groundwater level contour map, the potential map, the seepage gradient, and the seepage discharge of the three-dimensional seepage field under different working conditions. Based on the results of the three-dimensional flow field of the Hami dam, we presented an engineering application case of the IGA–UECM and suggestions for optimizing the grouting curtain. The main conclusions are as follows:

1. The standard GA algorithm is improved by making a new genetic operation to overcome the premature convergence shortage of the standard GA in the hydraulic conductivity inversion problem and is more efficient compared to the BO and TEP algorithms. When using this method to invert other dams’ hydraulic conductivity, it will be necessary to adjust the number of copies of excellent individuals according to the total number of samples to adapt to different sample sizes.
2. A new method and code for the inversion of dam foundation hydraulic conductivity by coupling the improved GA and the UECM is proposed, which fully considers engineering practicability. The result of the IGA–UECM to calculate the dam seepage field is reasonable and is expected to be widely used in dam seepage inversion.
3. When the UECM is used to calculate the seepage field of a concrete-face rockfill dam, the range of calculation area should be adjusted according to the terrain. For example, the faults affecting the seepage around the dam should be considered in the model for dams with faults nearby.
4. For the Hami concrete-face rockfill dam, the seepage discharge of the grouting curtain designed layout is 26.13 L/s. Compared to the change in grouting curtain size, the construction quality of the curtain grouting has the most significant impact on the water head reduction, the seepage gradient, and the seepage discharge of the anti-seepage system of the dam. The seepage stability of the Hami dam can still be ensured when the hydraulic conductivity of the grouting curtain is 2.5 times larger, owing to the poor construction quality of the curtain grouting. During construction management and control, it will be necessary to prevent the hydraulic conductivity of the grouting curtain from growing beyond 2.5 times.
5. The grouting curtain should be designed to extend to the impermeable layer as far as possible to reduce the impact of grouting construction quality on the anti-seepage effect.

## Figures and Tables

**Table 1:** The range of the Hami dam foundation’s hydraulic conductivity.

Rock Layer	Number	Hydraulic Conductivity (cm/s)
10 Lu–100 Lu	*K*_1_ (highly weathered)	(0.5–10) × 10^−3^
	*K*_2_ (slightly weathered)	(1.0–10) × 10^−4^
3 Lu–10 Lu	*K*_3_	(1.0–9.0) × 10^−5^
1 Lu–3 Lu	*K*_4_	(0.5–5.0) × 10^−5^
Fault zone	*K*_5_	(1.0–10) × 10^−6^

The rock layer is labeled by the permeable rate range. Lu is a measure of permeable rate.

**Table 2:** Drilling water head *E_M_* in the geological exploration.

Number	*E_M_*_1_	*E_M_*_2_	*E_M_*_3_	*E_M_*_4_	*E_M_*_5_	*E_M_*_6_	*E_M_*_7_	*E_M_*_8_	*E_M_*_9_	*E_M_*_10_	*E_M_*_11_	*E_M_*_12_
*E* (m)	1825.8	1740.8	1752.0	1750.0	1738.1	1739.0	1717.0	1717.4	1716.2	1715.7	1716.3	1710.9
*x*	166.51	163.60	−29.35	15.25	184.49	69.93	−23.25	−81.72	61.15	125.10	3.59	−248.61
*y*	0.00	90.00	150.00	150.00	150.00	150.00	300.00	300.00	300.00	300.00	400.00	550.00

**Table 3:** Optimization calculation working conditions of the grouting curtain layout.

Condition	Description	
HM-1	Normal water level at 1761.00 m	The grouting curtain is arranged in the design scheme.
HM-2		The hydraulic conductivity of the grouting curtain is 2.5 times larger than that of the design scheme.
HM-3		The grouting curtain is 10 m deeper than the design scheme.
HM-4		The grouting curtain is 20 m deeper than the design scheme.
HM-5		The grouting curtain is extended by 20 m to the left and right banks.
HM-6		The grouting curtain is shortened by 20 m to the left and right banks.
HM-7	Dead water level at 1732 m	The grouting curtain is arranged in the design scheme.
HM-8		The hydraulic conductivity of the grouting curtain is 2.5 times larger than that of the design scheme.
HM-9		The grouting curtain is 10 m deeper than the design scheme.
HM-10		The grouting curtain is 20 m deeper than the design scheme.
HM-11		The grouting curtain is extended by 20 m to the left and right banks.
HMX-12		The grouting curtain is shortened by 20 m to the left and right banks.

**Table 4:** Hydraulic conductivity of the Hami dam foundation.

Rock Layer	Number	Hydraulic Conductivity (cm/s)
10 Lu–100 Lu	*K*1	4.5 × 10^−3^
	*K*2	6.4 × 10^−4^
3 Lu–10 Lu	*K*3	3.1 × 10^−5^
1 Lu–3 Lu	*K*4	2.2 × 10^−5^
Fault zone	*K*5	1.1 × 10^−6^

**Table 5:** Maximum average seepage gradient of the Hami concrete-face rockfill dam’s grouting curtain.

Working Condition	HM-1	HM-2	HM-3	HM-4	HM-5	HM-6	HM-7	HM-8	HM-9	HM-10	HM-11	HM-12
Maximum seepage gradient	7.14	6.66	7.25	7.31	7.58	7.11	3.76	3.26	3.81	3.86	3.91	3.45
Location	III	III	II	II	II	III	III	III	III	III	III	II

I refers to the starting position of the anti-seepage curtain of the left dam abutment near the groundwater surface. II refers to the starting position of the anti-seepage curtain of the right dam abutment near the groundwater surface. III refers to the top of the anti-seepage curtain under the central toe board of the riverbed.

## Data Availability

Not applicable.
